# Lidocaine Suppresses Viability and Migration of Human Breast Cancer Cells: TRPM7 as a Target for Some Breast Cancer Cell Lines

**DOI:** 10.3390/cancers13020234

**Published:** 2021-01-10

**Authors:** Hengrui Liu, James P. Dilger, Jun Lin

**Affiliations:** Department of Anesthesiology, Health Science Center, Stony Brook University, Stony Brook, NY 11794, USA; hengrui.liu1@stonybrookmedicine.edu (H.L.); james.dilger@stonybrookmedicine.edu (J.P.D.)

**Keywords:** breast cancer cells, HEK293, viability, migration, TRPM7

## Abstract

**Simple Summary:**

The local anesthetic lidocaine suppresses some cancer cell lines but the mechanism is unclear. Melastatin-like transient receptor potential 7 (TRPM7) ion channels play a role in cancer and may be a target for lidocaine. The aim of our study is to test the hypothesis that lidocaine affects the viability and migration of breast cancer cells by regulating TRPM7. We conducted several assays to measure viability, migration, and TRPM7 function in the presence of lidocaine. Our results showed that (a) lidocaine suppresses viability and migration of six types of breast cancer cells, but with different potency; (b) TRPM7 plays a role in mediating the effects of lidocaine on viability and migration of at least four of these breast cancer cell types. Our work contributes to the understanding of the effect of lidocaine on breast cancer cells and helps guide its potential clinical application in the surgical treatment of breast tumors.

**Abstract:**

Background: The local anesthetic lidocaine suppresses some cancer cell lines but the mechanism is unclear. The melastatin-like transient receptor potential 7 (TRPM7) ion channel is aberrantly expressed in some cancers and may play a role in the disease. Hence, we suggested that lidocaine affects the viability and migration of breast cancer cells by regulating TRPM7. Methods: We measured the effects of lidocaine on TRPM7 function in HEK293 with exogenous TRPM7 expression (HEK-M7) using whole-cell patch-clamp and fura-2AM-based quench assay. We measured the effect of lidocaine on TRPM7 function, cell viability, and migration in TRPM7 expressing human breast cancer cell lines using fura-2AM-based quench, MTT, and wound-healing assays respectively. We compared cell viability and migration of wild type HEK293 cells (WT-HEK) with HEK-M7 and wild type MDA-MB-231 (WT-231) with TRPM7 knockout MDA-MB-231 (KO-231). Results: Lidocaine (1–3 mM) inhibited the viability and migration of all of these breast cancer cell lines. Functional evidence for TRPM7 was confirmed in the MDA-MB-231, AU565, T47D, and MDA-MB-468 cell lines where lidocaine at 0.3–3 mM suppressed the TRPM7 function. Lidocaine preferentially suppressed viability and migration of HEK-M7 over WT-HEK and WT-231 over KO-231. Conclusions: Lidocaine differentially reduced the viability and migration of human breast cancer cell lines tested. TRPM7 is one of the potential targets for the effects of lidocaine on viability and migration in MDA-MB-231, AU565, T47D, and MDA-MB-468.

## 1. Introduction

Breast cancer is the most common malignant tumor in women worldwide with over 2 million new cases and over 600,000 deaths per year [1]. In 2019 in the US, there were more than 300,000 new breast cancer cases and more than 40,000 breast cancer deaths [2]. Anesthesia can affect the treatment of breast cancer [3,4,5]. Some retrospective studies suggest that the use of regional anesthesia improves clinical outcomes after cancer surgeries [6,7]. One of the most commonly used local anesthetics, lidocaine has direct effects on the viability and migration of breast cancer cells [8,9]. However, the mechanism underlying lidocaine’s effects on breast cancer cells is still unclear.

Multiple ion channels have been suggested to be critical for the development of cancers [10,11]. Among them, studies have found that a Ca^2+^, Mg^2+^, and Zn^2+^ permeable channel, the melastatin-like transient receptor potential 7 (TRPM7) channel, is aberrantly expressed and plays a role in cancers [12]. For example, TRPM7 is overexpressed and promotes cancer cell proliferation in bladder cancer [13]. TRPM7 regulation of intracellular Ca^2+^ homeostasis was proposed to associate with cancer development [14,15]. The migration of pancreatic cancer cells was reported to be regulated by TRPM7 by a Mg^2+^-dependent mechanism [16]. For breast cancer, an in vivo study showed that lidocaine decreased mouse 4T1 breast cancer cell migration after surgery [17]. An in vitro study showed that TRPM7 is associated with the growth of the human breast cancer cell line MCF-7 [18].

Lidocaine has been reported to affect functions of TRPMs including TRPM7 in kidney tissues [19]. A study revealed that lidocaine ameliorates zinc toxicity in neurons through suppressing TRPM7 currents [20]. The TRPM7 was thought to be a target of lidocaine in some types of cancer. For example, in glioma cells, TRPM7 mediated the inhibition of lidocaine toward cancer cell proliferation [21]. In the present study, we tested the role of TRPM7 as a lidocaine target for human breast cancer cells. A better understanding of underlying mechanisms may optimize the use of lidocaine in clinical breast cancer treatment.

## 2. Results

### 2.1. Lidocaine Reduced the Viability of Human Breast Cancer Cell Lines

**Figure 1:**
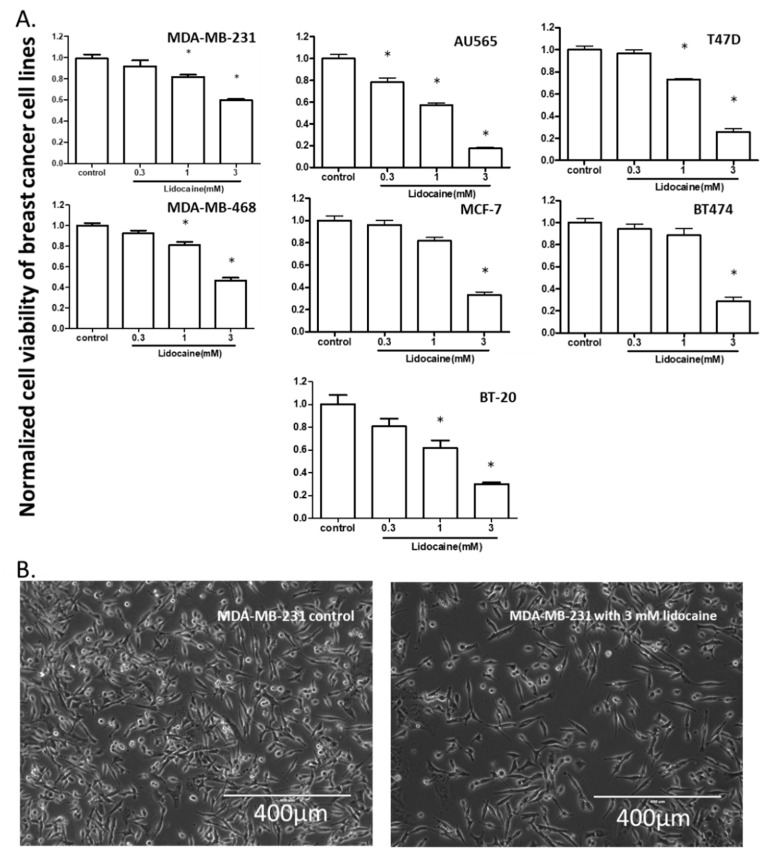
The effect of lidocaine on the viability of breast cancer cell lines. (**A**). Bar charts: [lidocaine] ≥ 0.3 mM inhibited AU565 viability, [lidocaine] ≥ 1 mM suppressed the viability of all cell lines except that [lidocaine] = 3 mM was needed for inhibition of BT474 and MCF-7 viability. “*” indicates the significant differences (*p* < 0.05) compared with the control. (**B**). Representative image of cells after lidocaine exposure (MDA-MB-231).

To test the effect of lidocaine on the viability of breast cancer cells, we exposed 7 human breast cancer cell lines (MDA-MB-231, AU565, T47D, MDA-MB-468, MCF-7, BT474, and BT-20) to 0.3, 1, and 3 mM of lidocaine and performed the MTT assay. A 24-h exposure to 3 mM lidocaine suppressed viability in all the cell lines. The viabilities of MDA-MB-231, AU565, T47D, MDA-MB-468, and BT-20 were all suppressed by 1 mM lidocaine, whereas at 0.3 mM lidocaine only inhibited the viability of AU565 (Figure 1).

### 2.2. Lidocaine Suppressed the Migration of Breast Cancer Cell Lines

**Figure 2:**
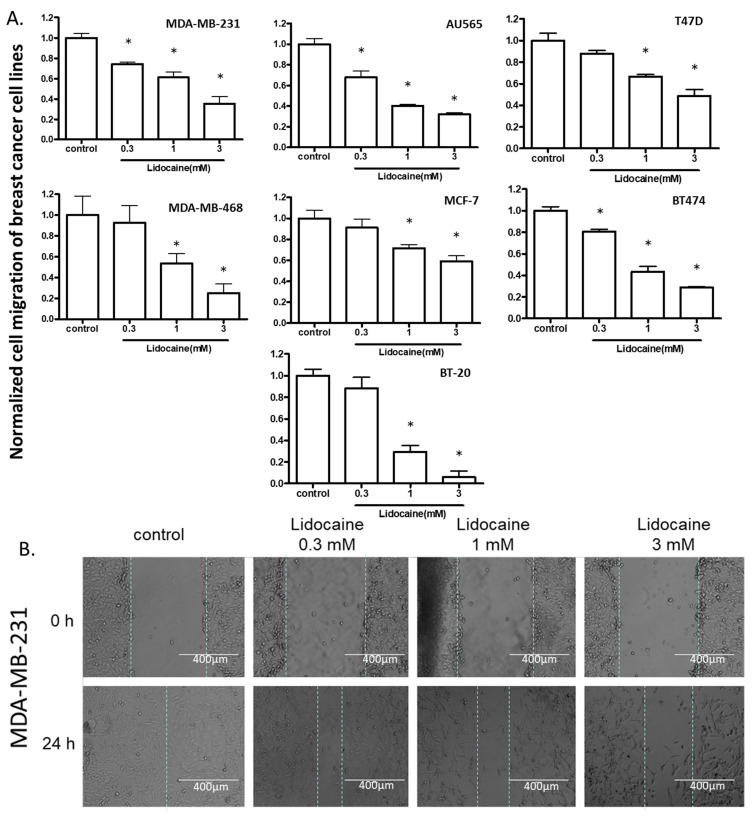
The effect of lidocaine on cell migration of breast cancer cell lines. (**A**). [lidocaine] ≥ 0.3 mM inhibited migration of MDA-MB-231, AU565, and BT474; [lidocaine] ≥ 1 mM suppressed the migration of all cell lines. “*” indicates the significant differences (*p* < 0.05) compared with the control. (**B**). Representative images (MDA-MB-231) from wound healing assay.

To test the effect of lidocaine on the migration of cells, we first created a wound on a monolayer of cells attached to a culture dish, treated the cells with 0.3, 1, or 3 mM lidocaine, and measured cell migration distance after 24 h. At 1 and 3 mM, lidocaine suppressed cell migration in all the cell lines, whereas, at 0.3 mM, migration of only MDA-MB-231, AU565, and BT474 was suppressed (Figure 2).

### 2.3. Lidocaine Suppressed the TRPM7 Channel Function in HEK293 Cells

**Figure 3:**
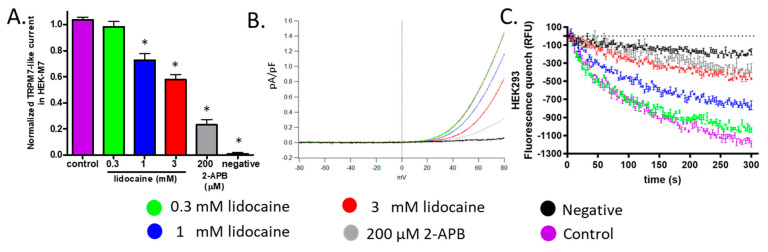
The effect of lidocaine on TRPM7 channels in HEK cells. (**A**). Patch-clamp assay. WT-HEK cells were used as a negative control for the cell model (black). 2-APB (200 µM) was used as a positive control (gray). [lidocaine] ≥ 1 mM suppressed TRPM7-like current in HEK-M7, *n* = 6, “*” indicates the significant differences (*p* < 0.05) compared with the control (purple). (**B**). Representative TRPM7-like current from patch-clamp. (**C**). Fluorescence quench assay: [lidocaine] ≥ 0.3 mM concentration-dependently decreased the influx of Mn^2+^ in HEK-M7 cells (*n* = 3). Current and quench data of control, negative control, and 200 µM 2-APB were published previously [22].

Because we expected that lidocaine affects cell viability and migration by regulating TRPM7 channels, we tested whether lidocaine blocks whole-cell patch-clamp currents through TRPM7 in HEK-M7 cells. Since, at physiological voltages, TRPM7 mediated currents are too small to be recorded, the currents were recorded with a voltage ramp protocol which reveals an outward current rectification (TRPM7 currents). 2-APB (200 µM), a nonselective TRPM7 inhibitor, blocked 80% of the TRPM7 currents at +80 mV. (Figure 3A,B gray) [22]. WT-HEK cells expressed low levels of TRPM7 and showed currents of less than 5 fA/pF (Figure 3A,B black) [22]. The TRPM7 currents in HEK-M7 were suppressed by lidocaine at 1 and 3 mM by 26% and 41% respectively. Lidocaine at 0.3 mM showed no significant effect compared with the control. (Figure 3A,B).

To confirm that inhibition of TRPM7 function by lidocaine is also present at physiological voltages, the fura-2AM-based fluorescence quench assay was used which monitors the influx of divalent cations (Mn^2+^) into the cell. Although, in WT-HEK, some fluorescence quenching was recorded at the endpoint of the assay (Figure 3C black), the quenching was much more significant in HEK-M7 (Figure 3C purple). The influx through WT-HEK cells represented a nonspecific influx of divalent cations. In HEK-M7 cells, the Mn^2+^ influx was dose-dependently inhibited by lidocaine at 0.3–3 mM (Figure 3C). The suppression of the TRPM7 function by lidocaine was quantitatively similar in both assays.

### 2.4. Lidocaine Suppressed the TRPM7 Function in Human Breast Cancer Cell Lines

We confirmed the expression of TRPM7 in all seven breast cancer cell lines used based on expression data from open-source mRNA sequencing (Figure S1A) and proteomics (Figure S2B) data. We used the fluorescence quench assay to determine the functional effect of lidocaine on TRPM7 in these cell lines. As the quenching depends on multiple effects including cell number, size of membrane area, intracellular Ca^2+^ concentration, loading efficiency, influx pathways, the quenching data cannot be used to compare TRPM7 expression levels among cell lines. We found that the quenching efficiency in some breast cancer cell lines was low (Figure S2A), thus, we focused on cell lines having a quench larger than 600 relative fluorescence units (RFU) (MDA-MB-231, AU565, T47D, and MDA-MB-468). Gd^2+^ was used as the negative control in the quenching assay, 10 µM was selected according to a concentration-dependent experiment (Figure S2B). In MDA-MB-231, AU565, T47D, and MDA-MB-468, 10 µM Gd^2+^ significantly suppressed the influx indicating a TRPM7-mediated mechanism, although the Gd^2+^ sensitive influx in MDA-MB-468 was relatively small.

**Figure 4:**
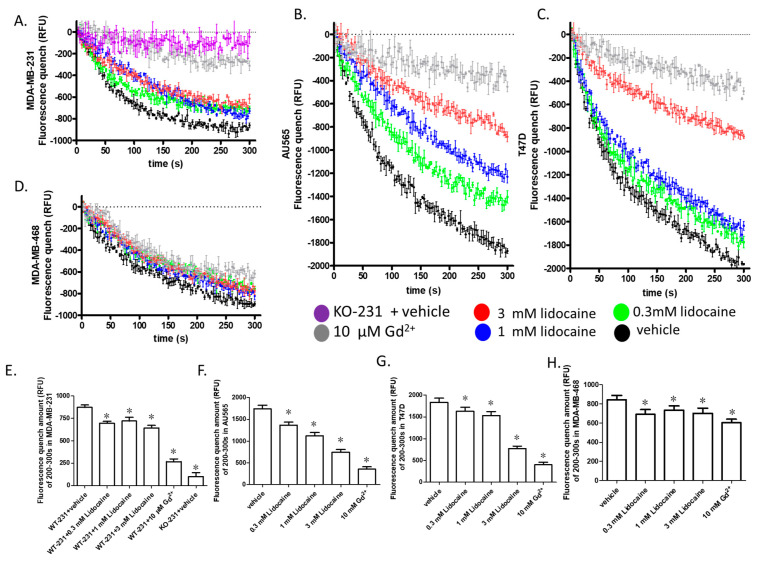
The effect of lidocaine on TRPM7 channels in breast cancer cell lines. (**A**–**D**). Fluorescence quench assay (*n* = 3): The fluorescence quenches followed by the addition of Mn^2+^ were plotted on the same scale. 10 µM Gd^2+^ was used as the negative control. (**E**–**H**). The average fluorescence quench amount of 200–300 s in MDA-MB-231, AU565, T47D, and MDA-MB-468. “*” indicates the significant differences (*p* < 0.05) compared with the control. The quench data of the vehicle, negative, and 200 µ 2-APB in MDA-MB-231 were published previously [22].

Lidocaine, at concentrations ≥0.3 mM, decreased the influx of Mn^2+^ in all of the four breast cancer cell lines tested (Figure 4A–D). The quench amount at the end of the assay (200–300 s) showed a dose-dependence in AU565 and T47D (Figure 4E–H). In MDA-MB-231, although the quench amount at the end of the assay (200–300 s) was not dose-dependent (Figure 4E), the initial rate of quench (determined as the slope of the linear regression of the first 100 s of data) was dose-dependent (−7.0, −5–5, −4.1 and −3.8 s^−1^ for 0, 0.3, 1 and 3 mM, respectively). However, in MDA-MB-468, the dose-dependence was not significant because of the small TRPM7-like influx.

### 2.5. TRPM7 Was a Potential Common Target of Lidocaine for Breast Cancer Cells

**Figure 5:**
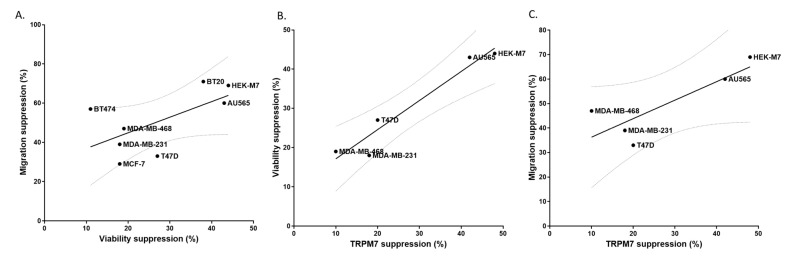
Correlations among the effects of 1 mM lidocaine on TRPM7 function, cell viability, and cell migration. (**A**). Suppression of migration vs. viability in 7 breast cancer cell lines and HEK-M7 cells (slope = 0.7954, r^2^ = 0.4016). (**B**). Suppression of viability vs. inhibition of TRPM7 function for HEK-M7 cells and the four breast cancer cells showing significant changes in fluorescence in the flux quenching assay (slope = 0.7426, r^2^ = 0.9351). (**C**). Suppression of migration vs. inhibition of TRPM7 function for HEK-M7 cells and the four breast cancer cells showing significant changes in fluorescence in the flux quenching assay (slope = 0.7563, r^2^ = 0.7047). TRPM7 function inhibition was calculated from the average of the quench fluorescence during the last 30 s. Linear regression was plotted and the 95% confidence band of the best fit line was shown.

Our data indicate a positive but relatively weak correlation between the ability of lidocaine to suppress migration vs. viability in human cancer cell lines (Figure 5A). This suggests a common mechanism underlying the effects of lidocaine on viability and migration. We calculated the TRPM7 inhibition percentage from the quench data to determine whether there was any correlation between TRPM7 inhibition and the results of the two cell assays. The resulting plots show a strong correlation between TRPM7 suppression and viability (Figure 5B) and a weaker one between TRPM7 suppression and migration (Figure 5C). This supports a role for TRPM7 as a potential up-stream target of lidocaine in the viability and migration of breast cancer cells.

### 2.6. TRPM7 Expression Increased the Sensitivity of HEK293 Cells to Lidocaine

**Figure 6:**
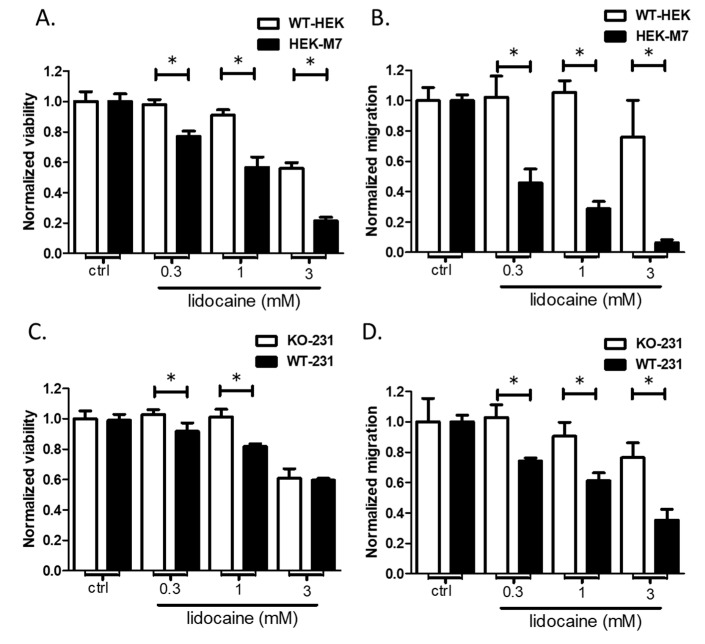
TRPM7 is necessary for the suppression of viability and migration by 0.3 and 1 mM lidocaine. Viability (**A**) and migration (**B**) of HEK cells containing very low levels of TRPM7 (WT-HEK) are unaffected by low concentrations of lidocaine. In contrast, viability and migration of HEK-M7 cells, in which TRPM7 is overexpressed, are suppressed. Viability (**C**) and migration (**D**) of MDA-MB-231 cells in which TRPM7 has been knocked out (KO-231) are not affected by low concentrations of lidocaine. In contrast, viability and migration of the wild-type cell line (WT-231, which express TRPM7) are suppressed. “*” indicates the significant differences (*p* < 0.05).

To further test the hypothesis that lidocaine affects the viability and migration cells via TRPM7, we compared the effects of lidocaine on WT-HEK cells (very low expression of TRPM7) with that of HEK-M7 cells (overexpression of TRPM7). Lidocaine preferentially suppressed the viability of HEK-M7 over WT-HEK. Although 3 mM lidocaine suppressed the viabilities of both cell lines, the lower concentrations of lidocaine suppressed the viability of HEK-M7 (Figure 6A). The same situation was observed for inhibition of cell migration by lidocaine. Migration of HEK-M7 cells was suppressed in a dose-dependent manner by lidocaine, but suppression of WT_HEK cell migration occurred only at 3 mM lidocaine (Figure 6B). These results suggest that TRPM7 is involved in the actions of lidocaine on the viability and migration of HEK cells.

### 2.7. Knockout of TRPM7 Decreased the Sensitivity of MDA-MB-231 Cells to Lidocaine

To test this hypothesis in a breast cancer cell line, we compared the effect of lidocaine on viability and migration of WT-231 and KO-231. The results paralleled the observations made on HEK cells. Viability (Figure 6C) and migration (Figure 6D) of the cell line lacking TRPM7, KO-231, were insensitive to 0.3 and 1 mM lidocaine. In contrast, the cell line containing TRPM7, WT-231, exhibited suppression of viability and migration at all concentrations of lidocaine. These results suggest that TRPM7 is involved in the actions of lidocaine on viability and migration of MDA-MB-231 cells.

## 3. Discussion

Lidocaine is one of the most commonly used local anesthetics in the clinic. It has been reported that lidocaine suppresses the viability and migration of many types of cancers [23]. In the present study, we focused on breast cancer using seven human breast cell lines to explore a potential target underlying the effect of lidocaine. Previous studies suggested that lidocaine blocks TRPM7 channels [20]. We demonstrated that under our experimental conditions, the function of the TRPM7 channel was suppressed by acute exposure to lidocaine. In HEK-M7, the TRPM7 current was suppressed by lidocaine at 1 and 3 mM by 26% and 41% respectively (Figure 3A,B). The fluorescence quench assay also revealed that TRPM7 channels were suppressed by lidocaine (Figure 3C). A previous study, using a different patch-clamp protocol, showed that lidocaine inhibited TRPM7 current with an IC_50_ of 11 mM [20]. These results are quantitatively similar to ours.

One challenge in the TRPM7 study is that no inhibitor has been shown to be ideally specific for TRPM7 [22]. We used 10 µM Gd^2+^ as the negative control in the fluorescence quench assay, but Gd^2+^ was not a TRPM7 specific inhibitor. This brings some uncertainty to the interpretation of the results. This uncertainty is reduced in our experiments with MDA-MB-231 by using knockout cells as a negative control (Figure 4A). Lidocaine decreased the Mn^2+^ influx by about 20% in MDA-MB-231. Our previous study showed that TRPM7 knockout resulted in slower growth of MDA-MB-231, but the cell numbers showed a significant difference only after 72 h [22]. In addition, although TRPM7 was reported to mediate breast cancer cell migration and invasion [24], we found that knockout of TRPM7 did not significantly suppress the migration of MDA-MB-231 after 24 h (Figure S3). This suggests that the chronic lack of TRPM7 function may be largely compensated by other mechanisms. A previous study showed that the suppression of TRPM7 by inhibitor 2-APB or Gin Rd significantly decreased cell viability [22]. Lidocaine preferentially suppressed the viability and migration of HEK-M7 over WT-HEK, suggesting that TRPM7 was involved in lidocaine effects (Figure 6A). The role of TRPM7 in the lidocaine effect was confirmed in breast cancer cell line MDA-MB-231: the effect of lidocaine (0.3–1 mM) on viability and migration was reduced by the knockout of TRPM7 (Figure 6C,D). Nevertheless, at 3 mM, lidocaine suppressed the viabilities of both MDA-MB-231 cell lines, implying that, at this concentration, lidocaine affects the viability of MDA-MB-231 cells through additional mechanisms.

The TRPM7 gene was expressed in all of the seven human breast cancer cell lines we used (Figure 1). In the viability and migration assay, we found that lidocaine has a common suppression effect in all breast cancer cell lines (Figure 1 and Figure 2). The general correlation between viability suppression and migration suppression suggested a common mechanism of lidocaine (Figure 5A). The functional data supported TRPM7 as a potential common upstream target of lidocaine (Figure 5B,C), for both viability and migration regulation. However, this evidence is correlative and the underlying mechanism remains unclear. A potential mechanism involves the effect of TRPM7 on epithelial-mesenchymal transition. TRPM7 was shown to affect cancer through its regulation of the epithelial-mesenchymal transition in prostate cancer [21], ovarian cancer [25], and colorectal cancer [26]. Since the epithelial-mesenchymal transition plays a role in breast cancer cell proliferation and migration [27], it might serve as a potential mechanism underlying the cancer pharmacology of lidocaine. In addition, although TRPM7 regulates both viability and migration of some breast cancer cells, it might act through different pathways. TRPM7 was reported to regulate breast cancer cell migration through its kinase domain [28,29]. However, the effect of the TRPM7 kinase domain on its channel function is still unclear [30].

Our results showed that (a) lidocaine suppresses viability and migration of all breast cancer cell lines with different potency; (b) TRPM7 is a target for the effects of lidocaine on viability and migration of MDA-MB-231; (c) data of AU565, T47D, and MDA-MB-468 supports the hypothesis, but not as strongly. Future studies are required to explore common mechanisms underlying the effects of lidocaine. Our work contributes to the understanding of the clinical effect of lidocaine on breast cancer treatment and is valuable for the optimization of clinical lidocaine application.

## 4. Materials and Methods

### 4.1. Cell Lines

HEK293 human kidney embryo cell line (WT-HEK), breast cancer cell lines MDA-MB-231 (WT-231), AU565, T47D, MDA-MB-468, MCF-7, BT474, and BT-20 were purchased from ATCC^®^ (Manassas, VA, USA). HEK-M7, HEK293 clone with the tetracycline-inducible murine TRPM7 gene (GenBankTM accession number AF376052) was a gift from Dr. Loren Runnels, Robert Wood Johnson Medical School (Piscataway, NJ, USA) [31]. TRPM7 knock-out MDA-MB-231 cell line (KO-231) was obtained from GenScript (Piscataway, NJ, USA) through GenCRISPRTM Technology [22].

### 4.2. Cell Culture

Dulbecco’s Modified Eagle Media (DMEM) with 10 mM piperazineethanesulfonic acid (HEPES), 10% Fetal bovine serum (FBS), and 2% pen/strep was used to culture WT-HEK, HEK-M7, MDA-MB-231, MDA-MB-468, MCF-7, BT474, and BT-20. Roswell Park Memorial Institute (RPMI)-1640 with 10 mM HEPES, 10% FBS, and 2% pen/strep was used to culture AU565 and T47D. A 37 °C, 5% CO_2_ humidified incubator was used to culture the cells.

### 4.3. Drugs

Generic Equivalent to Xylocaine^®^ (preservative free lidocaine HCl, 1%, 10 mg/mL parenteral solution) was purchased from Hospira Inc. (Lake Forest, IL, USA). The lidocaine was diluted into the culture medium accordingly. GdCl_2_, tetracycline, and 2-Aminoethyl diphenylborinate (2-APB) were purchased from Sigma-Aldrich (St. Louis, MO, USA), while 3-(4,5-Dimethylthiazol-2-yl)-2,5-diphenyltetrazolium bromide (MTT) and Fura-2-acetoxymethyl ester (fura-2AM) were purchased from Abcam (Cambridge, UK).

### 4.4. Viability Assay

Cell viability was determined by MTT assay [9]. Cells were cultured in 96-well plates in serum-free medium for 24 h before experiments. After the exposure to lidocaine for 24 h, 20 μL of 5 mg/mL MTT was added to cells. After 2 h of incubation, the resulting formazan crystals were dissolved in 200 μL dimethyl sulfoxide. A Multiskan™ FC Microplate Photometer (Waltham, MA, USA) was used to measure the absorbance at 490 nm. All the data were calibrated with the control data.

### 4.5. Migration Assay

Cell migration was assessed using a wound-healing assay [9]. Cells were cultured in 6-well plates and allowed to grow until reaching 95% confluency. A “wound” was created by scratching the cell monolayer with a 200-μL pipet tip. Then the cells were cultured with lidocaine in serum-free medium for 24 h. Images of wounds were taken at 0 and 24 h. The migration distance was analyzed with Image J. Each experimental condition was repeated at least three times.

### 4.6. Whole-Cell Patch-Clamping

Whole-cell patch-clamping was used to measure TRPM7 currents [22]. The PatchMaster software and EPC 10 Patch Clamp Amplifier (HEKA Elektronik, Berlin, Germany) were used to perform patch clamping. The SF-77B Perfusion Fast-Step Translator (Warner Instruments, Hamden, CT, USA) was used to apply and remove drugs. The bath solution (BS) contained (in mM) 140 NaCl, 10 HEPES, 1.3 CaCl_2_, 1.0 MgCl_2_, adjusted pH to 7.3 using KOH, 320–335 mOsm. Patch electrodes contained (in mM) 140 CsF, 7 NaCl, 10 HEPES, 11 EGTA, adjusted pH to 7.3 using CsOH, 300 mOsm. The whole-cell patch-clamp conFuration was created as described previously [22]. Capacitance and series resistance were compensated for the series resistance on the applied voltage. When recording the whole-cell currents, the voltage was clamped at −80 mV, then the voltage ramped from −80 to +80 mV. Currents were plotted as a function of voltage. At negative potentials, the whole-cell current is linear due to the seal resistance and/or voltage-independent channel activity. The current recorded at −80 to 0 mV was fitted to a line. The non-linear component over the entire voltage range was calibrated by subtracting this fit. This process revealed outward current rectification at positive potentials as expected for TRPM7 channels. The currents were then normalized by whole-cell capacitance.

### 4.7. Fura-2AM-Based Quench Assay

Fura-2AM-based quench assay was used to determined TRPM7 function [32]. Cells (5–6 × 10^4^ cells/well) were plated in 96-well plates. The culture medium was completely removed and replaced with fura-2AM loading-buffer (2 mM fura-2AM in BS). Following incubation (60 min at 37 °C), the loading buffer was removed, and the cells were washed once with BS before the addition of fresh BS as the assay buffer. The plates were then transferred to a 37 °C pre-warmed fluorescence plate reader (PerkinElmer Victor X3, San Jose, CA, USA). In the quench assay, the Ca^2+^-independent fluorescence of fura-2AM (excitation 360 nm; emission 510 nm) was monitored by the microplate photometer. After dye loading, cells were incubated with drugs for 5 min. After recording baseline fluorescence for 10 s, 10 mM MnCl_2_ was added and the fluorescence was recorded for 300 s. Fura-2 fluorescence decreases as Mn^2+^ enters the cells through TRPM7, displaces Ca^2+^ from the dye, and quenches the fluorescence. Each experiment was repeated three times.

### 4.8. Statistics

Means and standard deviations are shown in the figures. One-way ANOVA was used to assess significance (*p*  <  0.05). Dunnett’s post hoc tests were used to test differences between groups. GraphPad Prism (version 8, GraphPad Software, San Diego, CA, USA) was used to calculate statistics and plot the results.

## Data Availability

Data is contained within the article or Supplementary Material.

## Supplementary Materials

The following are available online at https://www.mdpi.com/2072-6694/13/2/234/s1, Figure S1: TRPM7 expression in breast cancer cell lines. A. The mRNA expression. The mRNA expression data (RNA seq) was downloaded and extracted from the Cancer Cell Line Encyclopedia (CCLE) [1]. B. The protein expression of TRPM7. The protein proteomics expression data was downloaded and extracted from Quantitative Proteomics of the Cancer Cell Line Encyclopedia [2], Figure S2: Fluorescence quench assay (*n* = 3). A. Fluorescence quench in Fura-2 loaded breast cancer cell lines (*n* = 3). The methods were described in the main text of the paper, *n* = 3. Cell lines with quench amounts larger than 600 relative fluorescence units (RFU) were used in the TRPM7 functional study. B. Effect of Gd2+ on TRPM7. WT-HEK was used as the negative control. [Gd2+] ≥ 0.05 µM concentration-dependently decreased the influx of Mn2+ in HEK-M7, Figure S3: The effect of TRPM7 knockout on cell migration of breast cancer cell line MDA-MB-231. A. Representative image from wound healing assay. B. The data were normalized by the division of the average migration distance of KO-231. 24 h after the creation of the wound, there was no significant difference in migration distance between KO-231 and WT-231 (*p* > 0.05).
