# Supplementary material for: Lidocaine Suppresses Viability and Migration of Human Breast Cancer Cells: TRPM7 as a Target for Some Breast Cancer Cell Lines
Source: Cancers (Basel). 2021 Jan 10;13(2):234. doi: 10.3390/cancers13020234 (PMC7827240; doi:10.3390/cancers13020234)
Supplement: Supplementary file 1 [file cancers-13-00234-s001.pdf]

Supplementary Materials

1. TRPM7 Expression in Breast Cancer Cell Lines

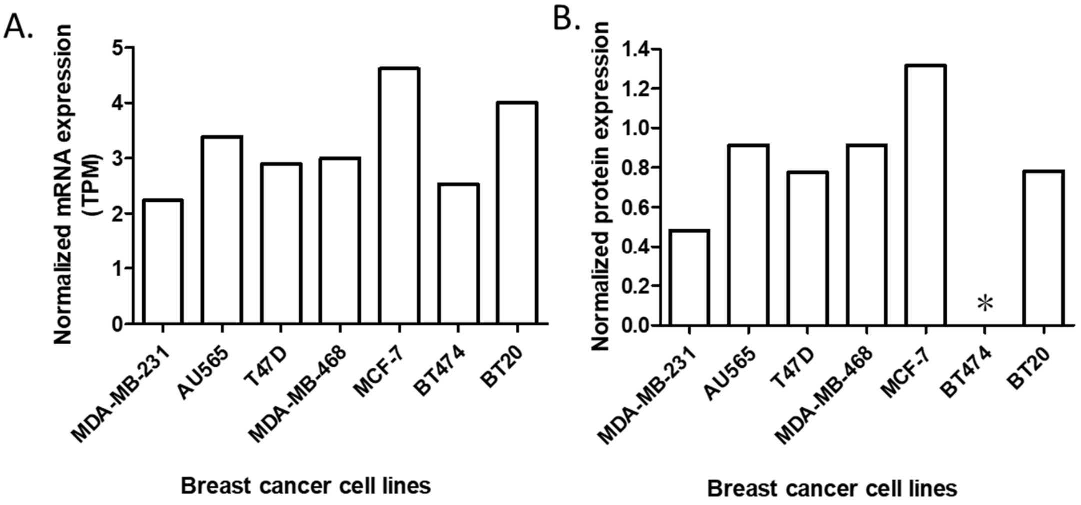

Figure S1. TRPM7 expression in breast cancer cell lines. **A.** The mRNA expression. The mRNA expression data (RNA seq) was downloaded and extracted from the Cancer Cell Line Encyclopedia (CCLE) [1]. **B.** The protein expression of TRPM7. The protein proteomics expression data was downloaded and extracted from the Quantitative Proteomics of the Cancer Cell Line Encyclopedia [2].

2. Additional Fluorescence Quench Data

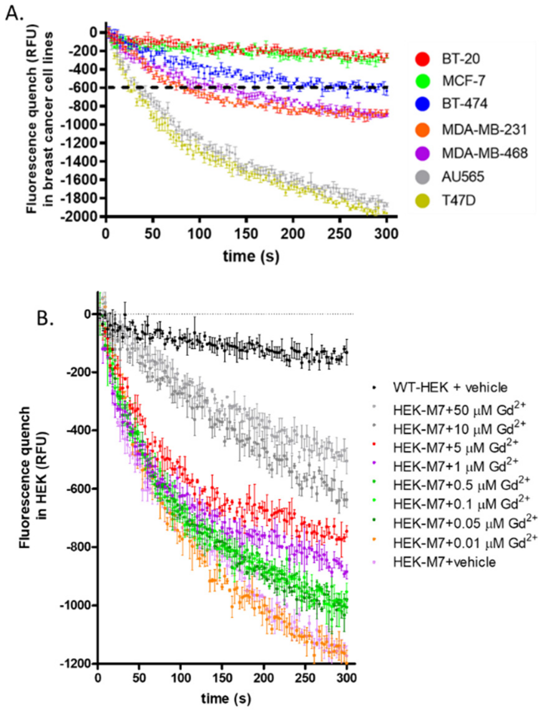

Figure S2. Fluorescence quench assay (n = 3). **A.** Fluorescence quench in Fura-2 loaded breast cancer cell lines (n = 3). The methods were described in the main text of the paper, n = 3. Cell lines with quench amounts larger than 600 relative fluorescence units (RFU) were used in the TRPM7 functional study. **B.** Effect of Gd^{2+} on TRPM7. WT-HEK was used as negative control. [Gd^{2+}] ≥ 0.05 μM concentration-dependently decreased the influx of Mn^{2+} in HEK-M7.

3. Effect of TRM7 Knockout on Migration of MDA-MB-231.

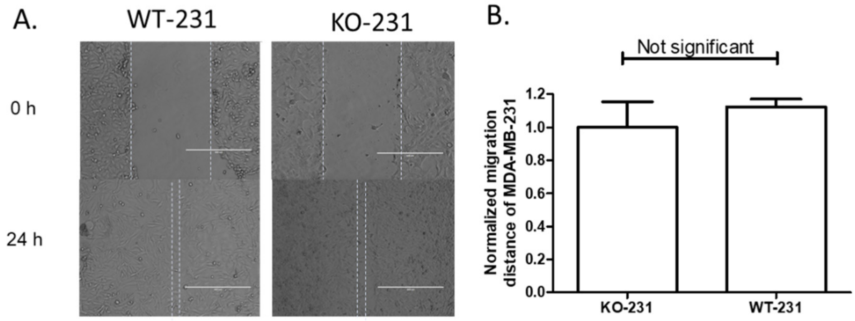

Figure S3. The effect of TRPM7 knockout on cell migration of breast cancer cell line MDA-MB-231. A. Representative image from wound healing assay. B. The data was normalized by the division of the average migration distance of KO-231. 24 hours after the creation of the wound, there was no significant difference in migration distance between KO-231 and WT-231 ($p > 0.05$).

References

1. Ghandi, M.; Huang, F.W.; Jané-Valbuena, J.; Kryukov, G.V.; Lo, C.C.; McDonald, E.R.; Barretina, J.; Gelfand, E.T.; Bielski, C.M.; Li, H.; et al. Next-generation characterization of the Cancer Cell Line Encyclopedia. *Nature* **2019**, *569*, 503-508, doi:10.1038/s41586-019-1186-3.
2. Nusinow, D.P.; Szpyt, J.; Ghandi, M.; Rose, C.M.; McDonald, E.R., 3rd; Kalocsay, M.; Jané-Valbuena, J.; Gelfand, E.; Schweppe, D.K.; Jedrychowski, M., et al. Quantitative Proteomics of the Cancer Cell Line Encyclopedia. *Cell* **2020**, *180*, 387-402.e316, doi:10.1016/j.cell.2019.12.023.
